# Molecular characterization of three *CYP450* genes reveals their role in withanolides formation and defense in *Withania somnifera*, the Indian Ginseng

**DOI:** 10.1038/s41598-022-05634-9

**Published:** 2022-01-31

**Authors:** H. B. Shilpashree, S. J. Sudharshan, Ajit K. Shasany, Dinesh A. Nagegowda

**Affiliations:** 1 Molecular Plant Biology and Biotechnology Lab, CSIR-Central Institute of Medicinal and Aromatic Plants, Research Centre, Allalasandra, GKVK Post, Bengaluru, 560065 India; 2 Plant Biotechnology Division, CSIR-Central Institute of Medicinal and Aromatic Plants, Lucknow, 226015 India

**Keywords:** Plant sciences, Plant molecular biology, Plant stress responses, Secondary metabolism

## Abstract

The medicinal properties of Ashwagandha (*Withania somnifera*) are attributed to triterpenoid steroidal lactones, withanolides, which are proposed to be derived from phytosterol pathway, through the action of cytochrome P450 (CYP450) enzymes. Here, we report the characterization of three transcriptome-mined *CYP450* genes (*WsCYP749B1, WsCYP76* and *WsCYP71B10*), which exhibited induced expression in response to methyl jasmonate treatment indicating their role in secondary metabolism. All three *WsCYP450s* had the highest expression in leaf compared to other tissues. *In planta* characterization of *WsCYP450s* through virus induced gene silencing (VIGS) and transient overexpression approaches and subsequent metabolite analysis indicated differential modulation in the accumulation of certain withanolides in *W. somnifera* leaves. While *WsCYP749B1*-vigs significantly enhanced withaferin A (~ 450%) and reduced withanolide A (~ 50%), its overexpression drastically led to enhanced withanolide A (> 250%) and withanolide B (> 200%) levels and reduced 12-deoxywithastramonolide (~ 60%). Whereas *WsCYP76-*vigs led to reduced withanolide A (~ 60%) and its overexpression increased withanolide A (~ 150%) and reduced 12-deoxywithastramonolide (~ 60%). Silencing and overexpression of *WsCYP71B10* resulted in significant reduction of withanolide B (~ 50%) and withanolide A (~ 60%), respectively. Further, while VIGS of *WsCYP450s* negatively affected the expression of pathogenesis-related (*PR*) genes and compromised tolerance to bacteria *P. syringae* DC3000, their overexpression in *W. somnifera* and transgenic tobacco led to improved tolerance to the bacteria. Overall, these results showed that the identified *WsCYP450s* have a role in one or several steps of withanolides biosynthetic pathway and are involved in conferring tolerance to biotic stress.

## Introduction

*Withania somnifera* (Ashwagandha, Indian ginseng or winter cherry) is a medicinal plant of high repute belonging to the Solanaceae family. The medicinal properties of this plant are attributed to a class of triterpenoid steroidal lactones collectively termed as withanolides. Plant extracts of *W. somnifera* and purified withanolides have demonstrated diverse pharmacological activities such as anti-inflammatory, anti-tumor, cardioprotective, neuroprotective and anti-bacterial properties^1,2^. Despite their immense pharmacological and therapeutic potential, commercial exploitation of withanolides has been limited owing to low *in planta* accumulation (in the range of 0.001–0.5% of DW) resulting in limited availability in purified forms^3^. Moreover, the accumulation of withanolides is influenced by various factors such as growth rate, tissue type, geographical and environmental conditions, and chemotype^4^. Moreover, successful metabolic engineering to improve withanolides production requires a proper understating of the genes involved in the biosynthetic pathway.

Withanolides are C_28_ steroids with an ergostane skeleton in which C_26_ and C_22_, or C_26_ and C_23_, are oxidized to form a δ- or γ-lactone^5^. Withanolides are derived through the universal phytosterol pathway, and it is proposed that the intermediates of phytosterol pathway derived via cycloartenol undergo various biochemical transformations such as hydroxylation and glycosylation postulated to be carried out by cytochrome P450 enzymes (CYP450s) and glycosyltransferases (GTs) leading to the biosynthesis of withanolides and withanosides, respectively^6–8^. CYP450 enzymes are a large class of heme-thiolate proteins that are ubiquitous in their presence across all genera of organisms and catalyze diverse reactions in pivotal molecular pathways. The CYP450 enzymes have been studied and classified by Nelson, DR (2009)^9^ and available on https://drnelson.uthsc.edu/CytochromeP450.html and an estimated 80 CYP450s have been assigned biochemical functions related to the plant triterpene metabolism^10^. Efforts have been made to identify and characterize CYP450s in *Withania somnifera*: Elicitor responsive *WsCYP98A* and *WsCYP76A* have been found to express abundantly in stalk and root, respectively, correlating positively to withanolides accumulation^11^. In another study, it was shown that WSCYP93Id protein catalyzed the conversion of withaferin A to an unidentified hydroxylated product^12^. Functional characterization of *WsCYP85A69* through miRNA and transient overexpression resulted in modulation of castasterone, stigmasterol and withanolides^13^. Further, it was shown that WsCYP85A69 catalyzed in vitro conversion of 6-deoxocastasterone into castasterone^13^. Despite some of these above studies, the knowledge on the *in planta* role of CYP450s in withanolides biosynthesis and defense is limited. Here, we present the molecular characterization of three genes encoding *CYP450s* and show the *in planta* involvement in withanolides biosynthesis and defense using silencing and overexpression studies.

## Materials and methods

All methods were carried out in accordance with relevant guidelines and regulations.

### Plant material, elicitor treatment and tissue collection

Seeds of Ashwagandha used in the study were obtained from our institutional gene bank. Ashwagandha variety (Poshita) developed by CSIR-Central Institute of Medicinal and Aromatic Plants (CSIR-CIMAP) was used in this study. Seeds were sown and plants were grown as described in Singh et al. (2015; 2017).

*Withania somnifera* leaves of 3^rd^ developmental stage were plucked from 10-week-old plants and kept in Petri dishes containing solutions of methyl jasmonate (MeJA; 500 mM) with respective controls. Samples were harvested at different time (h) intervals and stored at −80 °C until further use. For tissue-specific expression analysis berries, flowers, leaves, roots and stems were collected from field-grown *W. somnifera* plants^14,15^.

### Phylogenetic analysis

CYP450s belonging to respective families shown to be involved in secondary metabolism were used to construct the phylogenetic tree. Neighborhood joining method was used for tree construction using MEGAX with default setting and bootstrap value changed to 1000^16^.

### RNA isolation, cDNA synthesis and qRT-PCR analysis

Total RNA isolation, cDNA preparation and qRT-PCR were performed as reported earlier^14,15^. The cDNA was normalized using *18S* rRNA as endogenous control. qRT-PCR analysis was carried out with gene-specific primers (Table S1) that were designed outside the gene region used for cloning into pTRV2. The reaction was performed using 2X SYBR green mix (Thermo Scientific, USA) and run in StepOne Real-Time PCR System (Applied Biosystems, USA). The qRT-PCR conditions were: 94 °C for 10 min, followed by 40 cycles of 94 °C for 15 s and 60 °C for 45 s. Fold-change differences in gene expression were analyzed using the comparative cycle threshold (*C*_t_) method.

### Generation of VIGS and overexpression constructs

Plasmids pTRV1 and pTRV2^17^ procured from TAIR (www.arabidopsis.org) were used to generate VIGS constructs. A 500 bp gene-specific fragment was amplified from leaf cDNA using gene-specific primers (Table S1). The amplicon for each gene was cloned into a pGEMT-easy vector and sub-cloned into *Xba*I and *Xho*I sites of pTRV2 yielding pTRV2::*WsCYP450* constructs (Fig. S1). Next, the open reading frame (ORF) of each gene was PCR amplified using leaf cDNA and specific primers (Table S1). The amplicons were cloned into pJET1.2/vector and confirmed by nucleotide sequencing and subcloned into *Xba*I and *Sac*I sites of pBI121 vector to form pBI121::*WsCYP450* constructs (Fig. S1). The pTRV2- and pBI121- derived constructs were transformed into *A. tumefaciens* by freeze–thaw method.

### VIGS and transient overexpression of *WsCYP450s*

VIGS of *WsCYP450s* was performed as described by Singh et al.^14^. Infiltration was performed using a 1:1 ratio of *Agrobacteria* cultures harboring TRV1 and TRV2 or its derivatives using a 1-mL needleless syringe on the abaxial side of leaves of 4-leaf-staged plants. Thirty days post-infiltration, tissues exhibiting viral infection phenotype were harvested and used for transcript and metabolite analyses. For transient overexpression, overnight grown *Agrobacterium* culture was pelleted by centrifugation and resuspended in infiltration buffer (50 mM MES, 2 mM Na_3_PO_4_, 0.5% glucose, and 100 µM acetosyringone with pH adjusted to 5.6) to an OD_600_ of 0.2 and incubated at 28 °C for 3–4 h. Leaf infiltration was performed as described above into the first pair leaves of 6–8 leaf-staged plants and placed in the dark. The infiltrated leaves were harvested after 48 h for further metabolite and transcript analysis.

### Extraction and analysis of withanolides

Withanolides extraction and analysis were performed following Singh et al.^14^. Twenty mg oven-dried (55 °C) leaf tissue was ground using 1-mL absolute methanol, sonicated and the supernatant was collected. The ground tissue was re-extracted twice and obtained methanolic extracts were pooled in a scintillation vial and evaporated. The dried residue was resuspended in 3-mL of 70% methanol. This was extracted thrice with 3-mL chloroform. The lower layer of a chloroform extract (9-mL) was decolorized using charcoal and dried. To the dried samples, 200 µL of 7:3 chloroform: methanol was added. Withanolides were analyzed using HPLC (Model: Nexera, Shimadzu, Kyoto, Japan) fitted with C-18 column as per the program described previously^14^. Withanolide standards were procured from Natural Remedies Pvt. Ltd. (Bangalore, India). The quantity of sample used for injection was 20 µL. Catharanthine was used as an internal standard. The area of individual withanolides was determined after normalizing with the peak area of the internal standard, catharanthine (Sigma-Aldrich).

### Bacterial growth curve assay

The model plant pathogen *P. syringae* pv. tomato DC3000 was cultured in 5 mL nutrient broth and grown overnight in an incubator shaker at 28 °C. The culture was centrifuged and the bacterial pellet was resuspended in 10 mM MgCl_2_ and the cell density was adjusted to OD_600_ = 0.002. Bacterial infiltration and CFU determination were performed according to our earlier reports^14,15^. 3 days after inoculation, leaf discs from infected and buffer infiltrated control plants were collected from the infiltration zone. Later the cfu/cm^2^ was determined by plating serial dilutions of leaf extracts on *Pseudomonas*-specific agar plates after homogenization with 10 mM MgCl_2_.

### Generation of transgenic tobacco

Tobacco was transformed using *A. tumefaciens* harboring pBI121 and pBI121-derived pBI121::*CYP450* constructs by leaf disc co-cultivation method as described in Singh et al.^15^. Transformed shoots regenerated on MS (Murashige & Skoog)^18^ selection medium supplemented with kanamycin (100 mg/L^−1^) were transferred to ½ MS rooting media. Plantlets with well-developed roots were hardened and PCR-screened positive lines were transferred to the growth chamber. T_1_ plants were PCR-screened and positive lines were used for transcript and bacterial growth analyses^15^.

### Statistical analysis

The statistical analysis was performed using GraphPad 9.2. The results are represented as mean ± standard error (SE). Error bars denote SE of results obtained from at least three independent experiments. One-way ANOVA (analysis of variance) was performed followed by Tukey’s comparison test. The *p*-value 0.12 was considered as not significant (ns), 0.033 as significant (*), 0.002 as moderately significant (**), and 0.001 as highly significant (***).

### Ethical approval

Appropriate permissions for use of Ashwagandha variety Poshita have been obtained from National Gene Bank, CSIR-CIMAP, Lucknow, India.

## Results and discussion

### Identification of *WsCYP450s* involved in secondary metabolism

In Ashwagandha, few previous studies have reported molecular and biochemical characterization of some CYP450s and suggested their possible involvement in withanolides biosynthesis. For example, two CYP450s (*WsCYP98A* and *WsCYP76A*) were found to be highly expressed in stalk and root, respectively, and their expression was positively correlated with the accumulation of withanolides^11^. Another CYP450, WsCYP93Id was shown to convert withaferin A to an unidentified hydroxylated product under in vitro conditions^12^. In a recent study, in vitro biochemical studies showed that WsCYP85A69 is involved in catalyzing the conversion of 6-deoxocastasterone to castasterone, and *in planta* studies modulated the accumulation of castasterone, stigmasterol, and withanolides^13^. Despite these studies, continued efforts to identify more genes is needed to characterize CYP450 families to elucidate the highly complex withanolides biosynthesis as the pathway may involve multiple CYP450s.

In this study, to identify WsCYP450s possibly involved in withanolides biosynthesis, Withanomics (www.im-crop.snu.ac.kr) database was searched resulting in 474 sequences annotated as CYP450. Screening of these 474 sequences resulted in a subgroup of 159 sequences belonging to CYP71, CYP76, CYP82, CYP90, CYP96, CYP716, and CYP87 families that have been proposed to be involved in the secondary metabolic pathways^19,20^. Among them, 32 sequences showed a significant leaf-specific FPKM (> 10) value. Further, candidates with identical locus and sequence identity were eliminated resulting in 14 gene sequences (Fig. S2). In addition, a search of the in-house generated *W. somnifera* transcriptome showed 157 sequences annotated as CYP450 in which 107 sequences were annotated to be involved in secondary metabolisms such as sesqui- and triterpene biosynthesis, alkaloid, brassinosteroid, and steroid biosynthesis. Finally, 17 gene sequences remained after removal of identical and partial length candidates, followed by cross -referencing with shortlisted sequences from the Withanomics database (Fig. S2).

### Expression profiling of *WsCYP450s*

**Figure 1:**
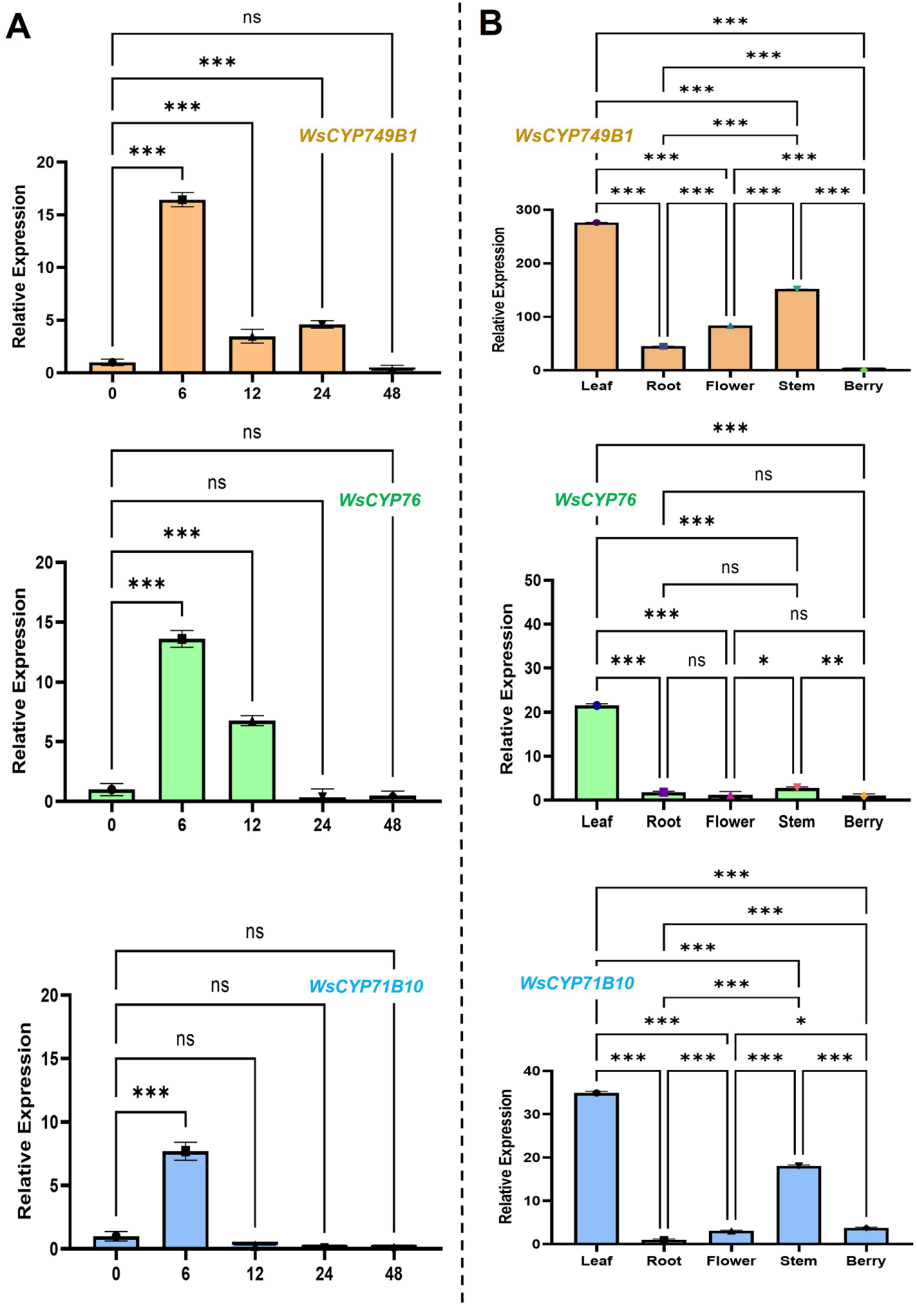
Analysis of transcript levels in response to MeJA and different tissues. (**A**) Effect of MeJA on the expression of *WsCYP749B1, WsCYP76,* and *WsCYP71B10* at time intervals of 0, 6, 12, 24, and 48 h after treatment. The expression is represented relative to the buffer control. (**B**) Relative transcript abundance of *WsCYP749B1, WsCYP76,* and *WsCYP71B10* in different tissues of *W. somnifera*. In each graph, the tissue having the least *Ct* was set to 1 to determine the relative abundance of transcripts in other tissues. *18s rRNA* was used as an internal reference for normalization. Error bars show mean ± SE of three independent experiments. Statistical significance represented as *p < 0.033, **p < 0.002, ***p < 0.001.

Generally, pathway genes involved in secondary metabolism including genes related to withanolides formation are induced in response to phytohormone elicitor MeJA with a corresponding increase of target secondary metabolites^15,21,21–23^. Hence, expression analysis of *WsCYP450* genes in leaves of MeJA treated plants was performed. This analysis showed that of the 17 analyzed genes, 8 genes were highly induced upon MeJA treatment. In this study, we have cloned and characterized 3 of the induced CYP450 candidates viz., *WsCYP749B1*, *WsCYP76* and *WsCYP71B10*. All three genes exhibited induced expression with maximum induction at 6 h upon exposure to MeJA (Fig. 1A). There was about a 14- and16-fold increase in the expression of *WsCYP76* and *WsCYP749B1*, respectively, with an eightfold increase in *WsCYP71B10* transcripts*.* After the peak induction at 6 h, while *WsCYP749B1* showed continued induction of ~four–fivefold at 12 h and 24 h post-MeJA treatment and reached basal level expression at 48 h, *WsCYP76* exhibited continued induction of sixfold at 12 h reaching basal level of expression thereafter (Fig. 1A). In contrast, *WsCYP71B10* exhibited induced expression only at 6 h and its expression remained at basal level in other time points (Fig. 1A). Similar to our results, two *CYP450* genes of *W. somnifera* (*WsCYP98A* and *WsCYP76A*) also showed induced expression in response to MeJA treatment with increased withanolide accumulation^11^. However, no *in planta* or functional studies were reported for these genes. In another study, WsCYP85A69 involved in castasterone formation also exhibited induced expression in response to MeJA and its transient silencing/overexpression in *W. somnifera* reduced/enhanced castasterone, stigmasterol and withanolides accumulation^24^. The observed MeJA-induced expression of *WsCYP749B1*, *WsCYP76* and *WsCYP71B10* in this study also indicates the involvement of these genes in secondary metabolism and possibly in withanolides biosynthesis.

Further, it was found that all three *WsCYP450s* exhibited a similar pattern of expression in different tissues with the highest expression in leaves compared to other tissues. Among the three candidates, *WsCYP749B1* showed highest expression in leaves followed by *WsCYP71B10* and *WsCYP76* (Fig. 1B). Both *WsCYP749B1* and *WsCYP71B10* transcripts exhibited siginificant differences in their expression pattern among all different tissues, whereas *WsCYP76* showed significant differences between only a few tissues. Moreover, with respect to tissues other than leaf, while *WsCYP749B1* showed comparatively higher expression in stem, flower and roots with least expression in berries, *WsCYP71B10* had higher levels of transcripts in stem followed by berry and flower with least expression in roots. In the case of *WsCYP76*, there was a negligible expression in all tissues other than leaves (Fig. 1B). Similarly, phytosterol pathway genes such as *WsHMGR*, *WsFPPS*, and *WsDWF5* showed differential expression in tissues with maximal expression in *W. somnifera* leaves^3,25^. In another study, expression level of obtusifoliol-14-demthylase (*CYP51*) and sterol methyl transferase (*SMT-1*) was shown to be preponderant in leaves of *W. somnifera* than in other tissues^26^. In our previous study, a transcription factor (WsWRKY1) regulating the biosynthesis of phytosterols and withanolides also exhibited higher expression in leaves^15^. It has been reported that withanolides are differentially present in different plant parts: however, some show tissue-specific accumulation. For instance, withaferin A is primarily synthesized in leaf tissue while withanolide A biosynthesis is seen specifically in roots^7^. Since CYP450s have the inherent ability to act on a range of substrates, their differential expression in tissues with maximal expression in leaves suggests that they may have a higher degree of involvement in the formation of leaf-specific withanolides. Whereas, they may have lesser role in the formation of withanolides specific to other tissues. The differential expression of *WsCYP450s* in tissues with maximal expression in leaves suggest that they may have a higher degree of involvement in formation of leaf-specific withanolides.

### Sequence analysis of *WsCYP405s*

**Figure 2:**
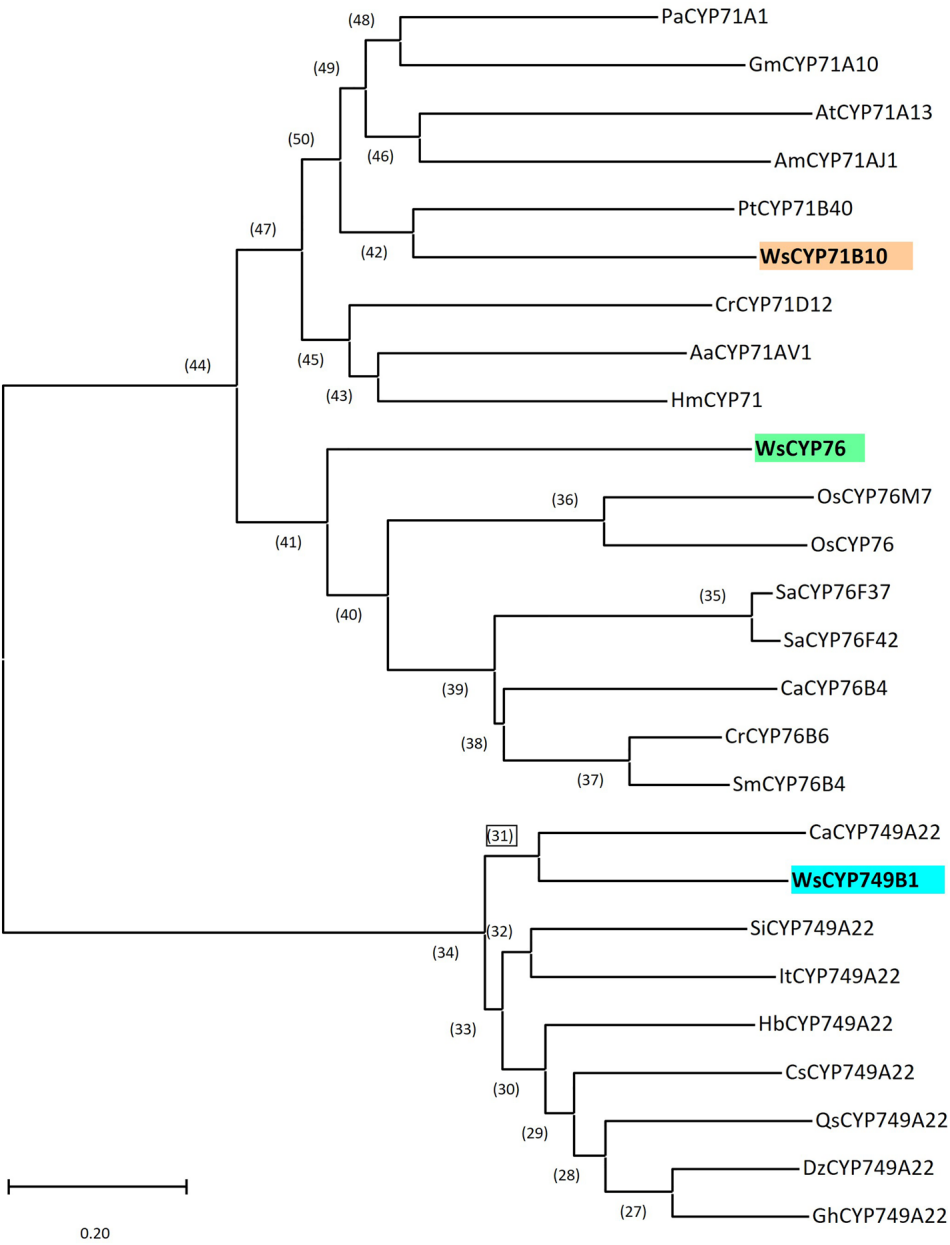
Phylogenetic relationship of WsCYP450s with other plant CYP450s involved in the plant metabolism. The tree was constructed using MEGA v.6, and the statistical reliability of individual nodes of the tree was assessed by bootstrap analyses with 1000 replicates. GenBank accessions of protein sequences used in the tree are listed in Table S2.

Analysis of confirmed sequences revealed that *WsCYP749B1*, *WsCYP76,* and *WsCYP71B10* contained open reading frames of 1533 bp, 1401 bp, and 1491 bp, respectively, that encode proteins of 511, 467, and 497 amino acids with a respective calculated molecular mass of 58.2 kDa, 53.19 kDa, and 56.61 kDa (accession numbers: MW298521, MW298522, and MW298523). Further analysis of amino acid sequences of the encoded proteins revealed the presence of [FW]-[SGNH]-x-[GD]-{F}-[RKHPT]-{P}-C-[LIVMFAP]-[GAD] consensus pattern for cysteine heme–iron ligand signature, where C is the heme iron ligand in all three WsCYP450s (Fig. S3). Phylogenetic analysis of all three WsCYP450s with CYP450s of other plants indicated that *WsCYP749B1*, *WsCYP76*, and *WsCYP71B10* fall in respective *CYP749*, *CYP71*, and *CYP76* families (Fig. 2). The percent identities of *WsCYP76* and *WsCYP71B10* were in the range of 34–41% and 37–48%, respectively, with other analyzed proteins of CYP76, and CYP71 families that have been shown to have a role in secondary metabolism. While WsCYP76 exhibited the highest (~ 41%) identity to geraniol 8-hydroxylase of *Catharanthus roseus*^27^ and *Swertia mussotii*^28^ followed by santalene/bergamotene oxidase (40%) of *Santalum album*^29^, WsCYP71B10 showed highest homology to *Populus trichocarpa* PtCYP71B40v3 involved in the conversion of aldoxime to defensive nitriles^30^, followed by ~ 45% and ~ 42% homology to *Persea Americana* trans-cinnamic acid 4-hydrolase (*PaCYP71A1)* and *Artemisia annua* amorpha-4,11-diene 12-monooxygenase (*AaCYP71AV1*), respectively^31,32^ (Fig. 2). Concerning *WsCYP749B1*, no enzymes for the CYP749 family (reported to be evolved only in Asteroids, Rosides and Ranunculales members) have been functionally characterized. However, the tomato *CYP749A20* gene was up-regulated in red and orange fruit with unknown functions^33^. *WsCYP749B1* exhibited ~ 48–56% homology to analyzed sequences with highest homology to *Capsicum annuum* CYP749A22 with unknown function of Solanaceae family. Further, prediction of subcellular localization using different tools indicated that all three WsCYP450s are membrane targeted, which is a characteristic of CYP450 (Table S3).

### VIGS of *WsCYP450s* modulates withanolides accumulation

**Figure 3:**
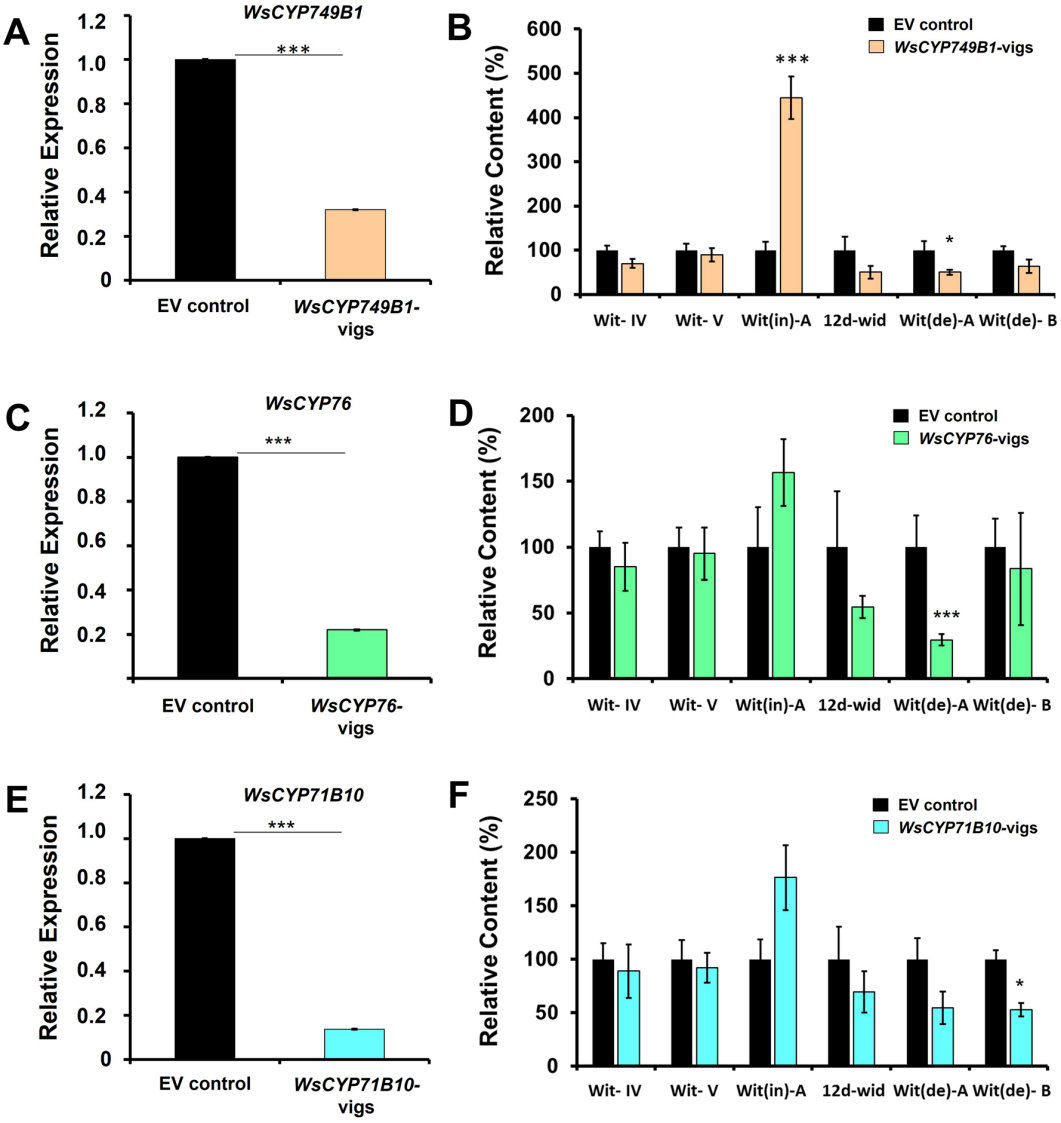
VIGS of *WsCYP450* genes and its effect on withanolides profile. Relative expression of *WsCYP450* genes (**A**, **C**, **E**) and contents of individual withanolides (**B**, **D**, **F**) in *W. somnifera* leaves infected with VIGS constructs and empty vector (EV) control samples. Expression levels of *WsCYP450s* were normalized to *18S rRNA* and are represented as expression relative to EV control that was set to 1. Quantity of withanolides is expressed relative to EV and peak area was determined after normalization with internal standard, catharanthine. Wit-IV, withanoside IV; Wit-V, withanoside V; Wit(de)-A, withanolide A; Wit(de)-B, withanolide B; Wit(in)-A, withaferin A; 12d-wit, 12-deoxywithastramonolide. Error bars show mean ± SE of at least three independent experiments. Statistical significance represented as *p < 0.033, **p < 0.002, ***p < 0.001, a significant difference.

VIGS approach has been successfully utilized for gene function studies in *W. somnifera*^3,14,15,25^. To verify the *in planta* role of *WSCYP749B1*, *WSCYP76* and *WSCYP71B10* in withanolide biosynthesis, VIGS was performed. As VIGS is prone to off target silencing^34^, the sequence region unique to each WsCYP450s was chosen to generate the silencing constructs. Thirty days post-infiltration leaves of similar developmental stages exhibiting typical viral infection symptoms were collected along with empty vector control for transcript and metabolite analysis. qRT-PCR analysis showed that the extent of silencing among the three CYP450 candidate genes ranged between 75 and 85% (Fig. 3). Among the three genes subjected to VIGS, *WsCYP71B10* exhibited maximum silencing effect (85%), followed by *WsCYP76* (80%), and *WsCYP749B1* (75%) (Fig. 3A,C,E). The degree of silencing of *WsCYP450s* in this study was comparable to the VIGS of other related genes of *W. somnifera* reported in earlier studies^3,14,15^. Subsequent analysis of withanolides through HPLC indicated that VIGS of all individual *WsCYP450s* exhibited modulation in the accumulation of one or more withanolides in comparison to control plants. Though VIGS of all three genes individually showed similar modulation profiles of withanolides, only withaferin A, withanolide A and withanolide B were significantly affected (Fig. 3B,D,F). While VIGS of *WsCYP749B1* significantly enhanced withaferin A content by ~ 450% and reduced withanolide A by 50% (Fig. 3B), silencing of *WsCYP76* and *WsCYP71B10* significantly decreased the accumulation of withanolide A (70%) and withanolide B (50%), respectively (Fig. 3D,F). The modulation in withanolide profile in VIGS samples indicated the functional role of *WsCYP749B1, WsCYP76* and *WsCYP71B10* genes in withanolide biosynthesis.

### Overexpression of *WsCYP450s* differentially affects withanolides accumulation

**Figure 4:**
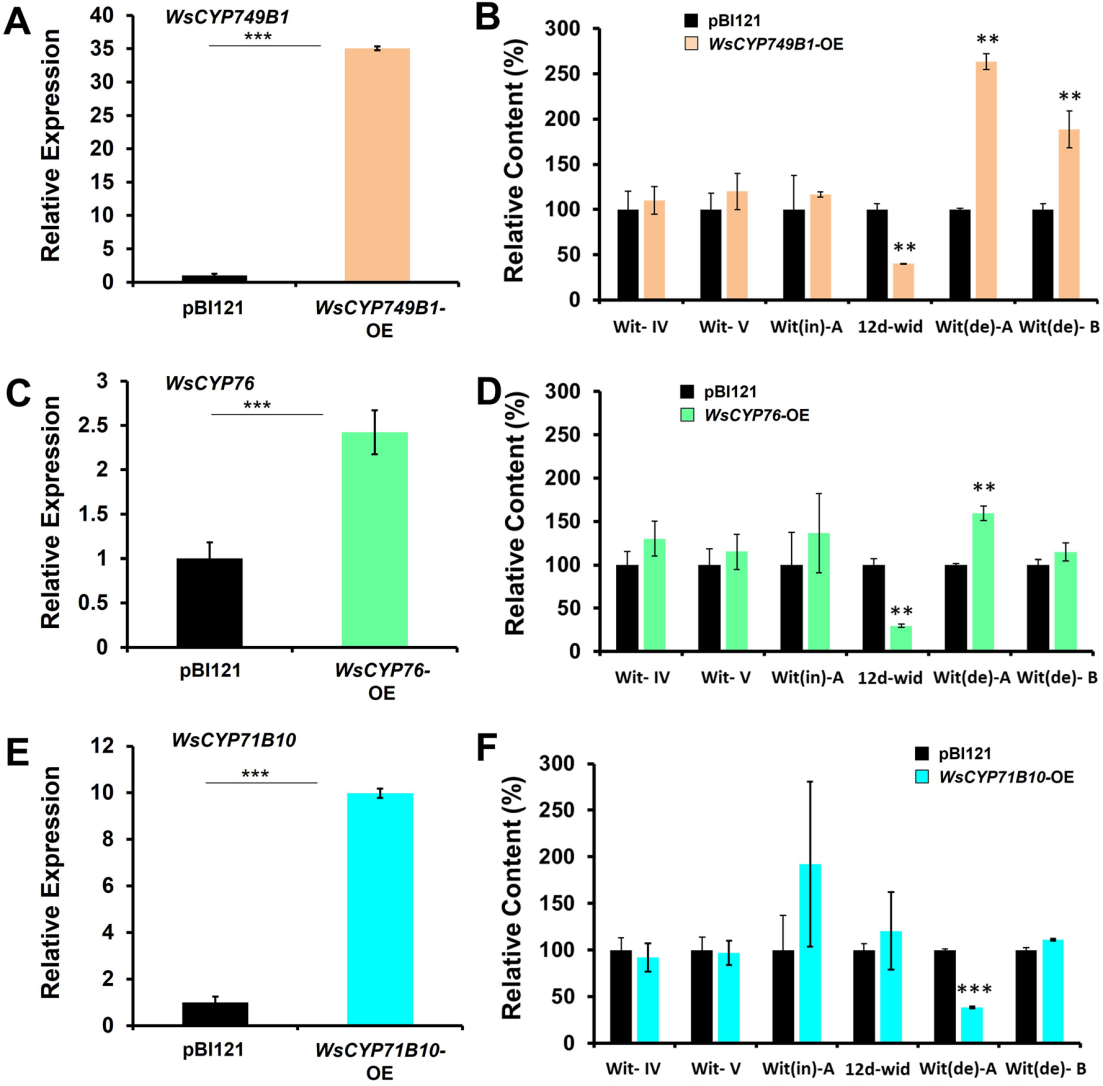
Transient overexpression of *WsCYP450* genes and its effect on withanolide accumulation. Relative expression of *WsCYP450* genes (**A**, **C**, **E**) and contents of individual withanolides (**B**, **D**, **F**) in *W. somnifera* leaves infiltrated with pBI121 and pBI121-derived *WsCYP450*-OE constructs. Expression levels of *WsCYP450s* were normalized to *18S rRNA* and are represented as expression relative to pBI121 control that was set to 1. Level of withanolides is expressed relative to pBI121 control and peak area was determined after normalization with internal standard, catharanthine. Wit-IV, withanoside IV; Wit-V, withanoside V; Wit(de)-A, withanolide A; Wit(de)-B, withanolide B; Wit(in)-A, withaferin A; 12d-wit, 12-deoxywithastramonolide. The results shown are from three (a) and six (b) experiments. Error bars show mean ± SE of three independent experiments. Statstical significance represented as *p < 0.033, **p < 0.002, ***p < 0.001, a significant difference.

To further investigate the effect of overexpression on withanolides accumulation, all three *WsCYP450* genes were individually overexpressed in *W. somnifera* leaves by Agroinfiltration. Analysis of gene expression by qRT-PCR showed a significant increase in the levels of transcripts ranging between 2.5- to 35-fold. *WsCYP749B1* had the highest expression (35-fold) followed by *WsCYP71B10* (10 fold) and *WsCYP76* (2.5-fold) compared to vector control samples (Fig. 4A,C,E). This increase in transcript levels of *WsCYP450s* resulted in differential accumulation of withanolides. Overexpression of *WsCYP749B1* and *WsCYP76* exhibited a similar accumulation profile with a significant increase of withanolide A by 250% and 150% and a drastic decrease of 12-deoxywithastramonolide by 60% and 70%, respectively (Fig. 4B,D). *WsCYP749B1* overexpression also lead to increased withanolide B content by 180% compared to control. Whereas, overexpression of *WsCYP71B10* significantly reduced (60%) withanolide A levels without affecting any other analyzed withanolides (Fig. 4F). There was a positive correlation with respect to transcript levels of *WsCYP749B1* and *WsCYP76,* and withanolide A content in VIGS and overexpression background (Figs. 3,4).

CYP450s have inherent ability to catalyze a diverse range of oxidative reactions exhibiting versatile substrate specificities and promiscuity. For instance, *A. annua* CYP71AV1 is involved in catalyzing three successive reactions in artemisinin pathway^35^. In *Salvia miltiorrhiza* the promiscuous nature of *CYP76AH3* and *CYP76AK1* bifurcate the tanshinones biosynthetic pathways and are involved in multiple steps suggesting it comprises a complex metabolic network^36^. Whereas *Coleus forskohlii* CYP76AH15 is involved in catalyzing reactions in two different pathways leading to ferruginol and forskolin^37^. In this study also, *in planta* silencing and overexpression of *WSCYP749B1*, *WSCYP76* and *WSCYP71B10* resulted in modulation in the accumulation of certain withanolides (Figs. 3, 4). From VIGS and overexpression results, it can be construed that the three characterized genes may be involved in one or several steps of the pathway though not necessarily in a sequential manner. While WSCYP749B1 may utilize withaferin A and 12-deoxywithastromonolide for formation of certain yet to be identified intermediates in the pathway, and could also have a role in formation of withanolide A and withanolide B. Likewise, WsCYP76 may be involved in utilizing 12-deoxywithastromonolide and could also have a role in withanolide A formation. Whereas WSCYP71B10 might have a role in utilizing withanolide A and forming withanolide B (Table S4).

### VIGS and overexpression of *WsCYP450s* affects *PR* genes and defense against *P. syringae*

**Figure 5:**
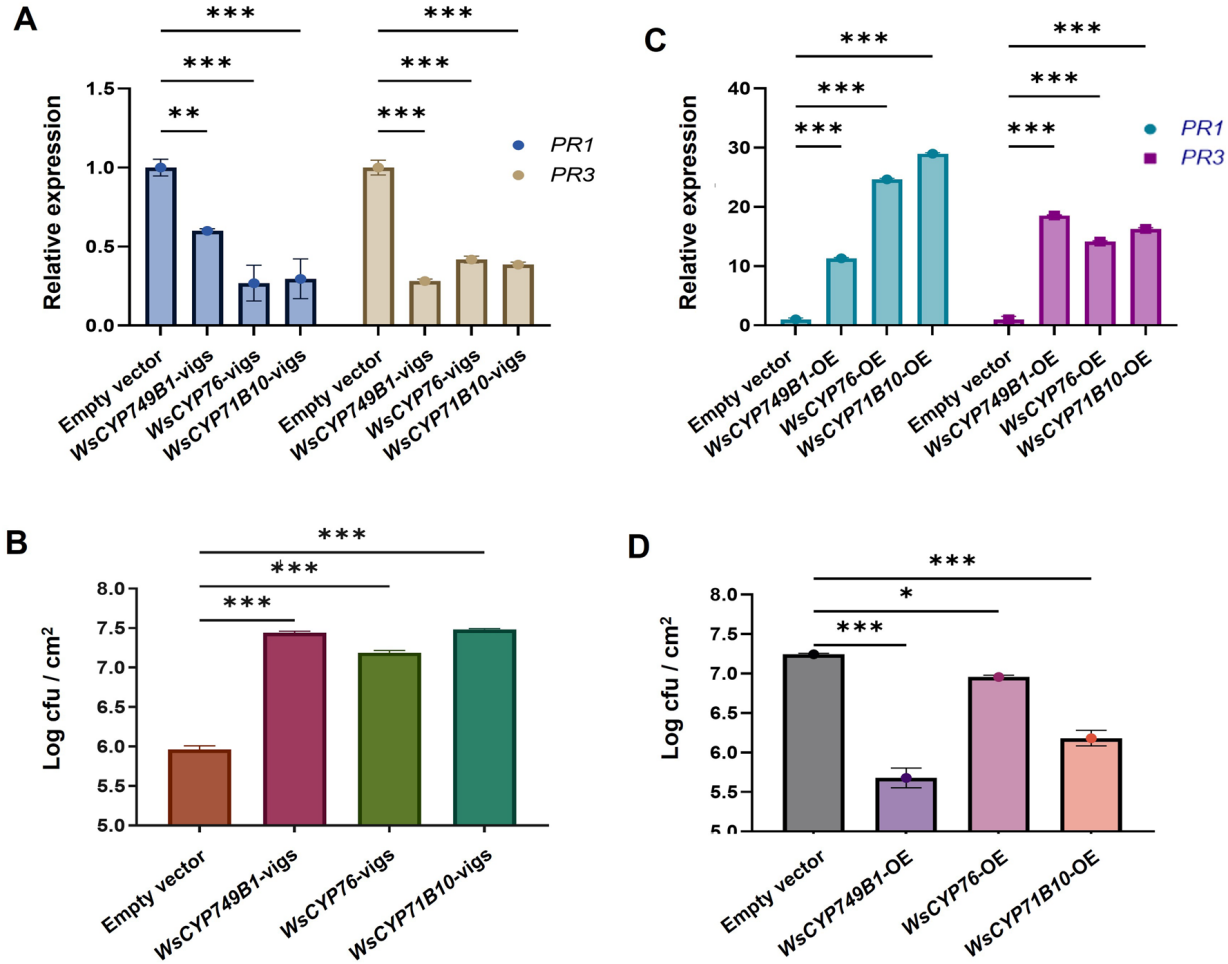
Effect of VIGS and overexpression of *WsCYP450s* on defense. Expression of *PR* genes in *WsCYP450s* silenced (**A**) and overexpressing (**C**) *W. somnifera* leaves. Expression levels of genes were normalized to *18S rRNA* and are represented as expression relative to the empty vector (EV) control, which was set to 1. *Pseudomonas syringae* growth assay in *WsCYP450s* silenced (**B**) and overexpressing (**D**) *W. somnifera* leaves. Growth assay was performed by infiltrating leaves of control and VIGS plants with *P. syringae* (DC3000). CFU was calculated at 3 dpi of *P. syringae* infiltration by plating serial dilutions of leaf disc extracts. Error bars show mean ± SE of three independent experiments. Statistical significance represented as *p < 0.033, **p < 0.002, ***p < 0.001, a significant difference.

In our previous studies in *W. somnifera*, it was observed that VIGS and overexpression of genes related to phytosterol and withanolides pathway modulate the expression of defense-related *PR* genes^14,15^. Moreover, withanolides have been shown to possess antibacterial and antifungal properties^38^. Since withanolides are derived from the phytosterol pathway and *WsCYP749B1*, *WsCYP76* and *WsCYP71B10* show their role in withanolides biosynthesis, we checked whether VIGS and overexpression of these genes have any effect on *PR* genes. Both salicylic acid (SA)- dependent *PR1* and jasmonate-dependent *PR3* exhibited a significantly reduced transcripts in all three *WsCYP450*-vigs samples in comparison to vector control. While *PR1* exhibited 40% downregulation in *WsCYP749B1-*vigs, and ~ 70–80% reduced transcripts in *WsCYP76-*vigs and *WsCYP71B10*-vigs samples, *PR3* downregulation was in the range of 60–70% in all three *WsCYP450*-vigs backgrounds (Fig. 5A).

CYP450s play important roles in plant defense through their involvement in phytoalexin biosynthesis, hormone metabolism and the biosynthesis of some other secondary metabolites^39^. As silencing and transient overexpression of *WsCYP450s* resulted in modulation of withanolides content and *PR* gene expression, bacterial growth assay was performed to determine *WsCYP450s*’ role in defense. Leaves from all three *WsCYP450-*vigs backgrounds developed severe disease symptoms and sustained more tissue damage than EV leaves when inoculated with *P. syringae* DC3000 (Fig. S4). Further, bacterial growth assay using extract isolated from inoculted leaves 3 dpi showed that *WsCYP450-*vigs samples exhibited adrastic increase in *P. syringae* DC3000 growth than that of EV control. The log cfu/cm^2^ for *WsCYP749B1*-vigs, *WsCYP76-*vigs and *WsCYP71B10-*vigs was found to be 7.4, 7.2 and 7.5, respectively, whereas it was ~ 6 cfu/cm^2^ for EV control (Fig. 5B).

In contrast to the effect of VIGS, overexpression of *WsCYP450s* led to upregulation of both *PR1* and *PR3* genes and enhanced the tolerance to bacterial growth. While *PR1* exhibited 10- to 30-fold enhanced expression, *PR3* showed ~ 14 to 18-fold increased expression in different *WsCYP450* overexpression samples in comparison to control (Fig. 5C). Further, leaves overexpressing *WsCYP450-*vigs remained healthy and exhibited no necrotic phenotype compared to control at 4 dpi of *P. syringae* DC3000. Subsequent bacterial growth assay revealed a significant reduction in the growth of *P. syringae* DC3000. The log cfu/cm^2^ in *WsCYP749B1*, *WsCYP76* and *WsCYP71B10* overexpressing tissue was 5.5, 6.9 and 6, respectively, in comparison to 7.2 in vector control. The observed phenotype and bacterial growth in *P. syringae* infiltrated samples correlated with elevated and reduced expression of *WsCYP749B1*, *WsCYP76* and *WsCYP71B10* (Fig. 5D). It is interesting to note that *PR* genes were affected in both VIGS and OE backgrounds indicating that the products (one or more withanolides) formed by *WsCYP749B1*, *WsCYP76* and *WsCYP71B10* enzymes could be linked to defense signaling. In our earlier studies also, silencing of *WsSQS* of phytosterol pathway and silencing and overexpression of *WsWRKY1* involved in regulation of phytosterol and withanolides formation, showed positive correlation with expression of PR genes^14,15^. Furthermore, AtCYP76C2 and AtCYP71A12 from *Arabidopsis* were found to be associated with defense mechanism against *P. syringae* infection^40,41^. Hence, it is clear that the three identified genes are involved in bacterial defense, however, how the change in expression of *WsCYP450s* affected *PR* gene expression is quite intriguing and interesting and needs further investigation. The enzymatic products of these genes (one or more withanolides) may play a role in signaling as well as providing tolerance through their antibacterial activity.

### Heterologous overexpression of *WsCYP450s *in transgenic tobacco confers tolerance to* P. syringae*

**Figure 6:**
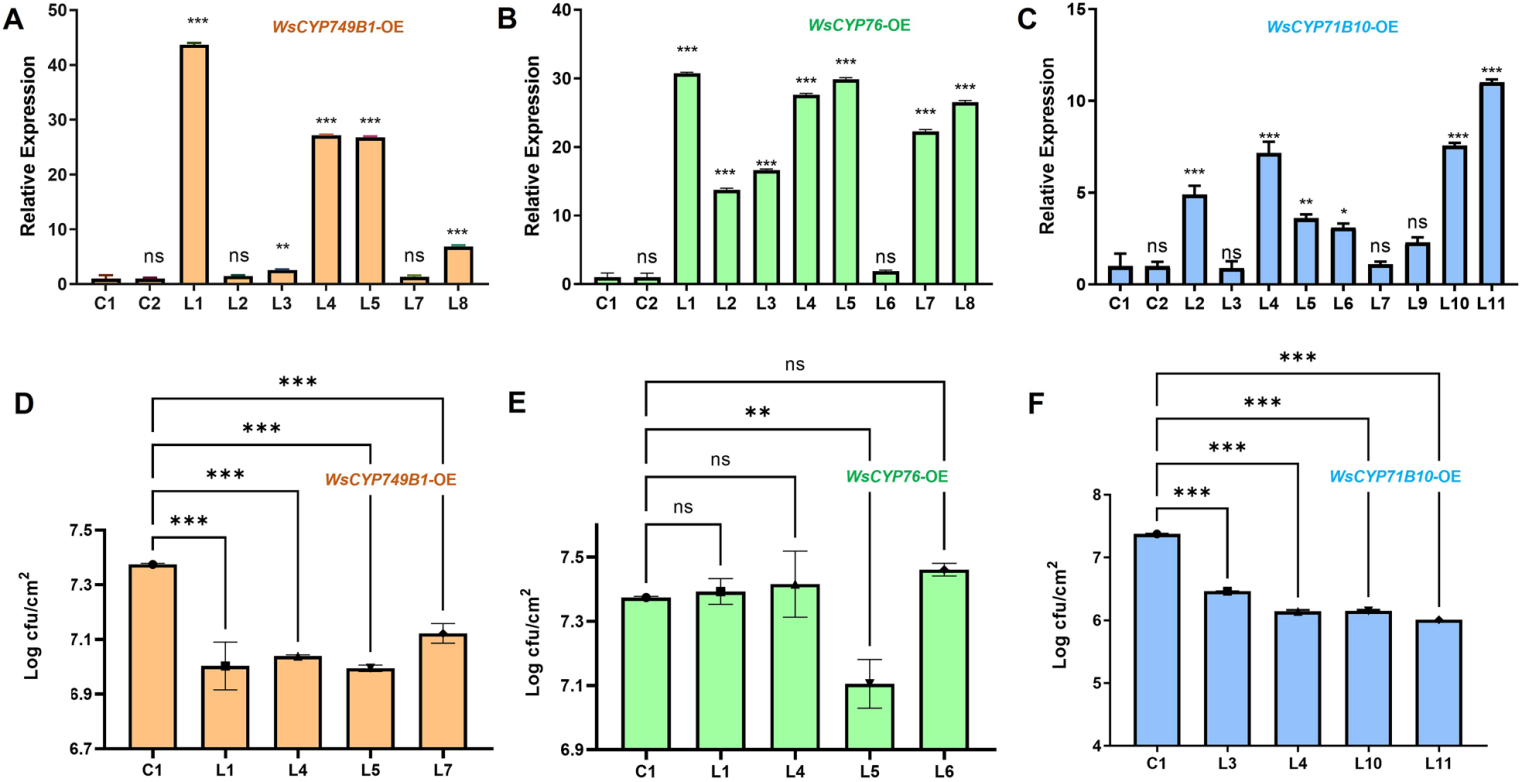
Analysis of gene expression and bacterial growth in transgenic tobacco overexpressing *WsCYP450s*. (**A**, **C**, **E**) Expression levels of *WsCYP450* genes in different transgenic tobacco lines. Expression levels of *WsCYP450s* were normalized to internal reference *NtEF1α* and arerepresented as expression relative to pBI121 control that was set to 1. (**B**, **D**, **F**) Bacterial growth assay with *P. syrinage* (DC3000) strains. Growth assay was performed by infiltrating leaves of control and transgenic *WsCYP450*-OE lines with *P. syringae* (DC3000). The bacterial growth (CFU) at 3 dpi was obtained by plating serial dilutions. Error bars show mean ± SE of three independent experiments. Statistical significance represented as *p < 0.033, **p < 0.002, ***p < 0.001, a significant difference.

Heterologous overexpression of plant CYP450s has been shown to confer tolerance to various stresses including tolerance against bacteria^42^. To further investigate the defensive role of *WsCYP450s* in a heterologous system, independent transgenic tobacco lines overexpressing *WsCYP450s* were generated (Fig. S5). Transcript abundance of respective genes in 7, 8 and 9 independent lines of *WsCYP749B1, WsCYP76* and *WsCYP71B10*, respectively, was determined by RT-qPCR. While the expression of *WsCYP749B1* in *WsCYP749B1-*OE tobacco lines ranged from 1- to 45-fold (Fig. 6A), the expression of *WsCYP76* and *WsCYP71B10* in *WsCYP76*-OE and *WsCYP71B10*-OE lines, ranged from 3- to 32-fold (Fig. 6B) and 1- to 12-fold (Fig. 6C), respectively. Three higher expressors along with one least expressor for each *WsCYP450* gene and control plants were chosen for bacterial growth assays. Analysis of bacterial growth using extracts isolated from leaf discs of *P. syringae* infiltrated leaves at 3 dpi revealed that higher *WsCYP450* overexpressing tobacco plants supported significantly lower multiplication of *P. syringae* compared to extracts from leaf discs of pBI121 plants (Fig. S6). While all overexpressing lines of *WsCYP749B1* and *WsCYP71B10* exhibited a significant reduction in bacterial growth (Fig. 6D,F), only one line of *WsCYP76* (line 9.5) showed a drastic decrease in bacterial growth (cfu ~ 7.1) (Fig. 6E). The degree of tolerance to bacterial growth in comparison to control plant was highest in *WsCYP71B10*-OE lines (cfu 6.0–6.46) followed by *WsCYP71B10-OE *lines (cfu 6.99–7.12) (Fig. 6). Similar to our results, overexpression of *Panax ginseng* *PgCYP76C9* conferred enhanced resistance to *P. syringae* in transgenic Arabidopsis^43^.

## Conclusion

In this study three MeJA-inducible *CYP450s* having higher expression in leaves were investigated for their *in planta* role using silencing and overexpression approaches. The results demonstrated that change in the expression of these genes by silencing or overexpression resulted in significant differential modulation of certain withanolides indicating their role in one or more steps of the withanolides biosynthetic pathway. In addition, our study showed that these three *WsCYP450s* have a role in defense and confer tolerance against bacteria. Overall, the study demonstrated the *in planta* role of *WsCYP749B1, WsCYP76* and *WsCYP71B10* in withanolides biosynthesis and defense against bacteria. Further biochemical characterization of these WsCYP450s could provide insights into their exact role in withanolides formation. The identified genes could also be utilized for metabolic engineering of *W. somnifera* for targeted enhancement of certain withanolides either in cell cultures or at the whole-plant level. Additionally, these genes could be utilized for generating plants tolerant to bacterial pathogens.

## Supplementary Information

Supplementary Information.

## References

[1] Sehgal N (2012). Withania somnifera reverses Alzheimer’s disease pathology by enhancing low-density lipoprotein receptor-related protein in liver. Proc. Natl. Acad. Sci. U. S. A..

[2] Mirjalili MH, Moyano E, Bonfill M, Cusido RM, Palazón J (2009). Steroidal lactones from withania somnifera, an ancient plant for novel medicine. Molecules.

[3] Agarwal AV (2018). Virus-induced silencing of key genes leads to differential impact on withanolide biosynthesis in the medicinal plant, *Withania somnifera*. Plant Cell Physiol..

[4] Dhar N (2013). Dynamics of withanolide biosynthesis in relation to temporal expression pattern of metabolic genes in *Withania somnifera* (L.) Dunal: a comparative study in two morpho-chemovariants. Mol. Biol. Rep..

[5] Chen LX, He H, Qiu F (2011). Natural withanolides: an overview. Nat. Prod. Rep..

[6] Sangwan RS (2008). Withanolide a is inherently de novo biosynthesized in roots of the medicinal plant ashwagandha (*Withania somnifera*). Physiol. Plant..

[7] Singh S (2014). Sterol partitioning by HMGR and DXR for routing intermediates toward withanolide biosynthesis. Physiol. Plant..

[8] Lockley WJS, Rees HH, Goodwin TW (1976). Biosynthesis of steroidal withanolides in Withania somnifera. Phytochemistry.

[9] Nelson DR (2009). The cytochrome P450 homepage. Hum. Genomics.

[10] Ghosh S (2017). Triterpene structural diversification by plant cytochrome P450 enzymes. Front. Plant Sci..

[11] Rana S (2014). Molecular characterization of two A-type P450s, WsCYP98A and WsCYP76A from *Withania somnifera* (L.) Dunal: expression analysis and withanolide accumulation in response to exogenous elicitations. BMC Biotechnol..

[12] Srivastava S (2015). Light and auxin responsive cytochrome P450s from *Withania somnifera* Dunal: cloning, expression and molecular modelling of two pairs of homologue genes with differential regulation. Protoplasma.

[13] Sharma A, Rather GA, Misra P, Dhar MK, Lattoo SK (2019). Gene silencing and over-expression studies in concurrence with promoter specific elicitations reveal the central role of Wscyp85a69 in biosynthesis of triterpenoids in *Withania somnifera* (L.) Dunal. Front. Plant Sci..

[14] Singh AK (2015). Virus-induced gene silencing of Withania somnifera squalene synthase negatively regulates sterol and defence-related genes resulting in reduced withanolides and biotic stress tolerance. Plant Biotechnol. J..

[15] Singh AK (2017). A WRKY transcription factor from *Withania somnifera* regulates triterpenoid withanolide accumulation and biotic stress tolerance through modulation of phytosterol and defense pathways. New Phytol..

[16] Tamura K, Stecher G, Peterson D, Filipski A, Kumar S (2013). MEGA6: molecular evolutionary genetics analysis version 6.0. Mol. Biol. Evol..

[17] Liu Y, Schiff M, Dinesh-Kumar SP (2002). Virus-induced gene silencing in tomato. Plant J..

[18] Murashige T, Skoog F (1962). A revised medium for rapid growth and bio assays with tobacco tissue cultures. Physiol. Plant..

[19] Moses T (2015). OSC2 and CYP716A14V2 catalyze the biosynthesis of triterpenoids for the cuticle of aerial organs of *Artemisia annua*. Plant Cell.

[20] Werck-Reichhart D, Bak S, Paquette S (2002). Cytochromes P450. Arab. B..

[21] Chen F, Ren CG, Zhou T, Wei YJ, Dai CC (2016). A novel exopolysaccharide elicitor from endophytic fungus *Gilmaniella* sp. AL12 on volatile oils accumulation in *Atractylodes lancea*. Sci. Rep..

[22] Bhat WW (2012). Molecular cloning, bacterial expression and promoter analysis of squalene synthase from *Withania somnifera* (L.) Dunal. Gene.

[23] Dhar N (2014). Cloning and functional characterization of three branch point oxidosqualene cyclases from *Withania somnifera* (L.) Dunal. J. Biol. Chem..

[24] Sharma A, Rather GA, Misra P, Dhar MK, Lattoo SK (2019). Jasmonate responsive transcription factor WsMYC2 regulates the biosynthesis of triterpenoid withanolides and phytosterol via key pathway genes in *Withania somnifera* (L.) Dunal. Plant Mol. Biol..

[25] Agarwal AV (2017). Comprehensive assessment of the genes involved in withanolide biosynthesis from *Withania somnifera*: chemotype-specific and elicitor-responsive expression. Funct. Integr. Genomics.

[26] Pal S (2011). Comparative withanolide profiles, gene isolation, and differential gene expression in the leaves and roots of *Withania somnifera*. J. Hortic. Sci. Biotechnol..

[27] Collu G (2001). Geraniol 10-hydroxylase, a cytochrome P450 enzyme involved in terpenoid indole alkaloid biosynthesis. FEBS Lett..

[28] Wang J (2010). Cloning and functional analysis of geraniol 10-hydroxylase, a cytochrome p450 from swertia mussotii Franch. Biosci. Biotechnol. Biochem..

[29] Diaz-Chavez ML (2013). Biosynthesis of sandalwood oil: *Santalum album* CYP76F cytochromes P450 produce santalols and bergamotol. PLoS ONE.

[30] Irmisch S, Jiang Y, Chen F, Gershenzon J, Köllner TG (2014). Terpene synthases and their contribution to herbivore-induced volatile emission in western balsam poplar (*Populus trichocarpa*). BMC Plant Biol..

[31] O’Keefe DP, Leto KJ (1989). Cytochrome P-450 from the mesocarp of avocado (*Persea americana*). Plant Physiol..

[32] Komori A (2013). Comparative functional analysis of CYP71AV1 natural variants reveals an important residue for the successive oxidation of amorpha-4,11-diene. FEBS Lett..

[33] Vasav AP, Barvkar VT (2019). Phylogenomic analysis of cytochrome P450 multigene family and their differential expression analysis in *Solanum lycopersicum* L. suggested tissue specific promoters. BMC Genomics.

[34] Dinesh-Kumar SP, Anandalakshmi R, Marathe R, Schiff M, Liu Y (2003). Virus-induced gene silencing. Methods Mol. Biol..

[35] Ro DK (2006). Production of the antimalarial drug precursor artemisinic acid in engineered yeast. Nature.

[36] Guo Y (2015). Identification and functional analysis of a cytochrome P450 gene CYP9AQ2 involved in deltamethrin detoxification from *Locusta migratoria*. Pestic. Biochem. Physiol..

[37] Pateraki I (2017). Total biosynthesis of the cyclic AMP booster forskolin from *Coleus forskohlii*. Elife.

[38] Iqbal Choudhary M (1995). Antifungal steroidal lactones from Withania coagulance. Phytochemistry.

[39] Xu J, Wang XY, Guo WZ (2015). The cytochrome P450 superfamily: key players in plant development and defense. J. Integr. Agric..

[40] Godiard L (1998). CYP76C2, an Arabidopsis thaliana cytochrome P450 gene expressed during hypersensitive and developmental cell death. FEBS Lett..

[41] Kempthorne CJ (2021). Metabolite profiling reveals a role for intercellular dihydrocamalexic acid in the response of mature *Arabidopsis thaliana* to *Pseudomonas syringae*. Phytochemistry.

[42] Pandian BA, Sathishraj R, Djanaguiraman M, Prasad PVV, Jugulam M (2020). Role of cytochrome P450 enzymes in plant stress response. Antioxidants.

[43] Balusamy SRD, Rahimi S, Cho YG, Senthil K, Yang DC (2017). Overexpression of geraniol 10-hydroxylase from Panax *ginseng* conferred enhanced resistance to *Pseudomonas syringae* in Arabidopsis. Plant Growth Regul..

