# Supplementary material for: Molecular characterization of three CYP450 genes reveals their role in withanolides formation and defense in Withania somnifera, the Indian Ginseng
Source: Sci Rep. 2022 Jan 31;12:1602. doi: 10.1038/s41598-022-05634-9 (PMC8803918; doi:10.1038/s41598-022-05634-9)
Supplement: Supplementary file 1 — Supplementary Information. [file 41598_2022_5634_MOESM1_ESM.pdf]

Supporting information

Molecular characterization of three *CYP450* genes reveals their role in withanolides formation and defense in *Withania somnifera*, the Indian Ginseng

H.B. Shipashree[1], S.J. Sudharshan[1], Ajit K. Shasany[2], and Dinesh A. Nagegowda[1]*

*Corresponding author: da.nagegowda@cimap.res.in

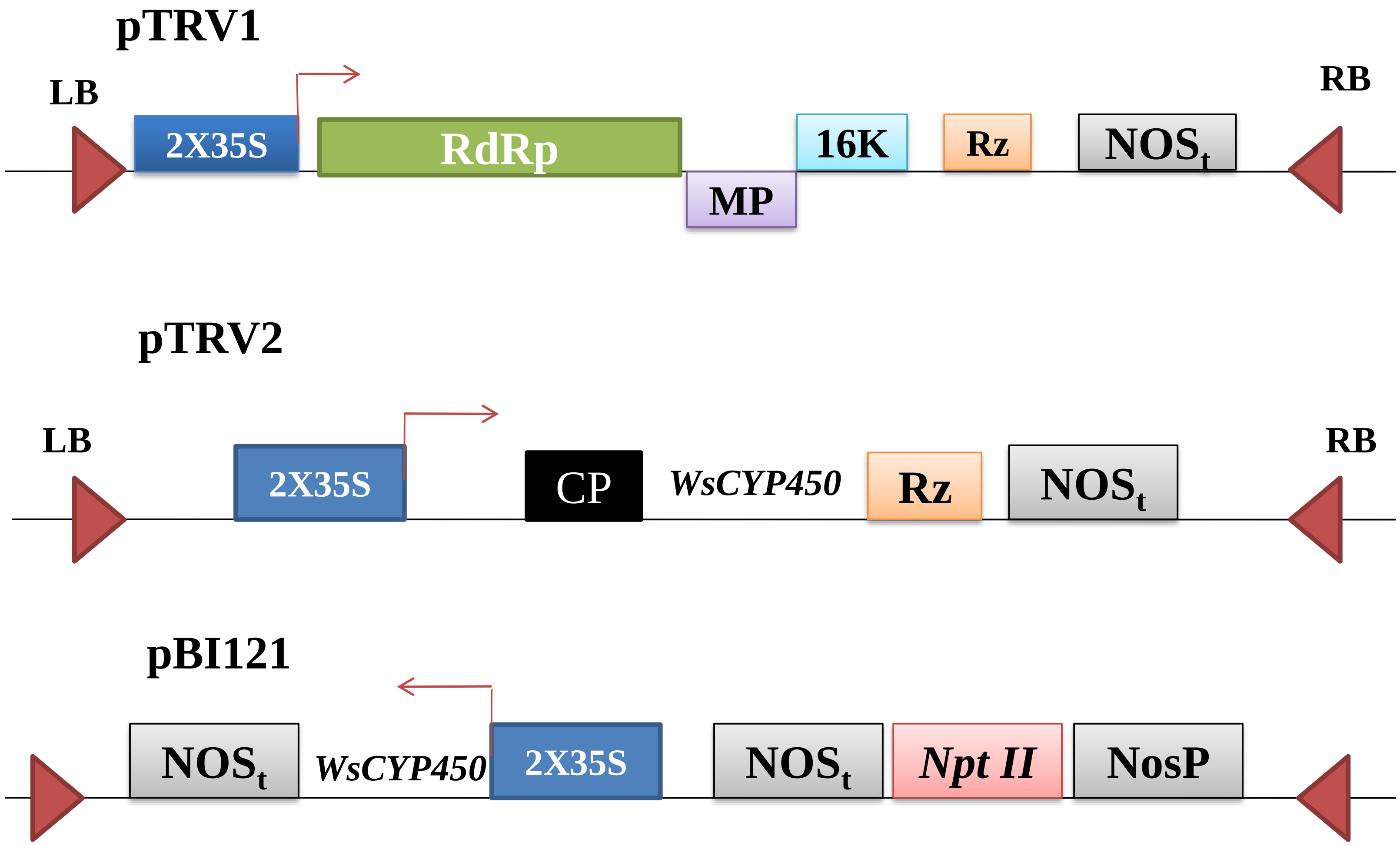

Fig. S1: pTRV1, and pTRV2- and pBI121- derived vector maps used for silencing and overexpression studies in *Withania somnifera* and *Nicotiana tabacum*.

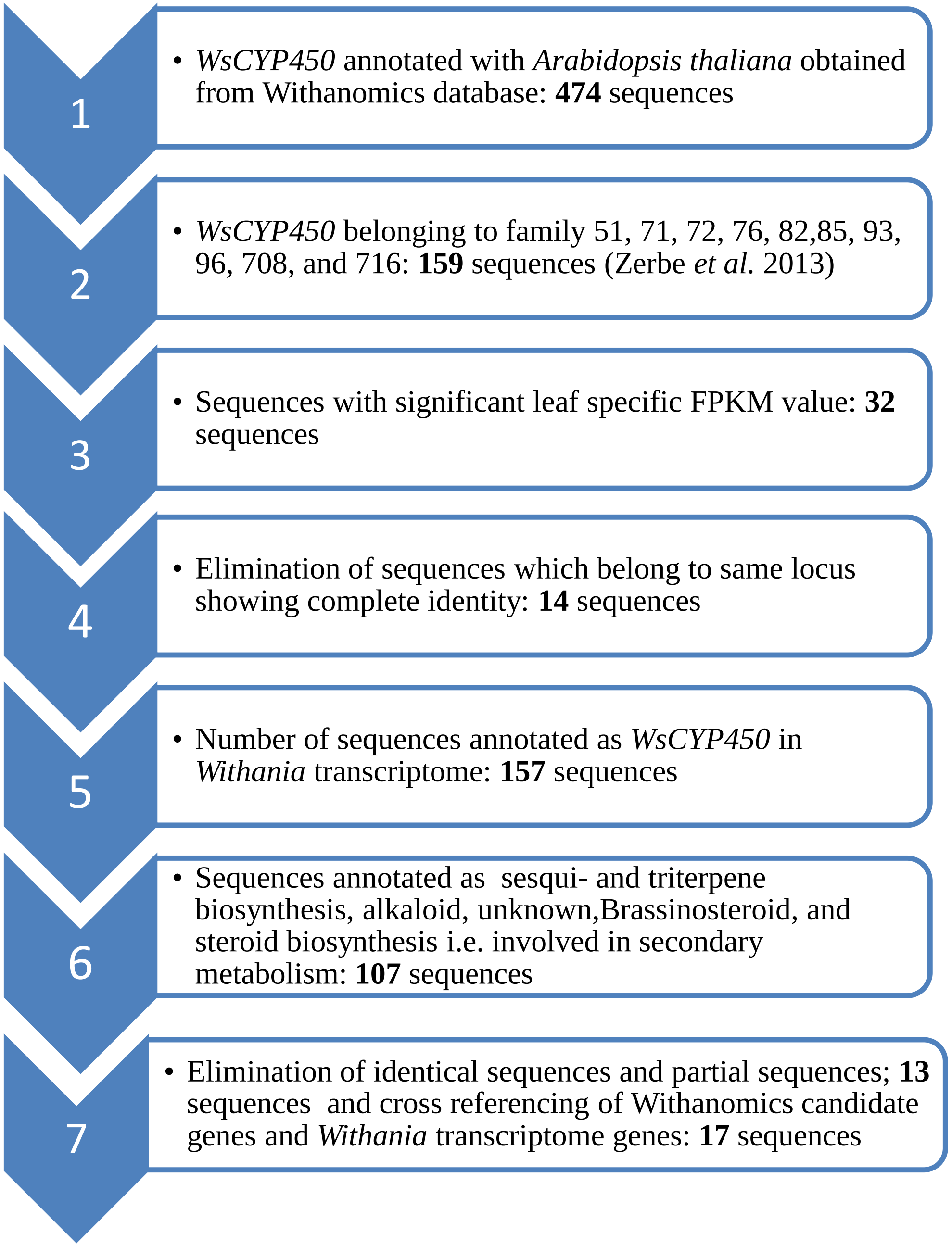

Figure S2: Flow-chart showing **s**election of putative *Cytochrome P450s* from *W. somnifera* transcriptome database

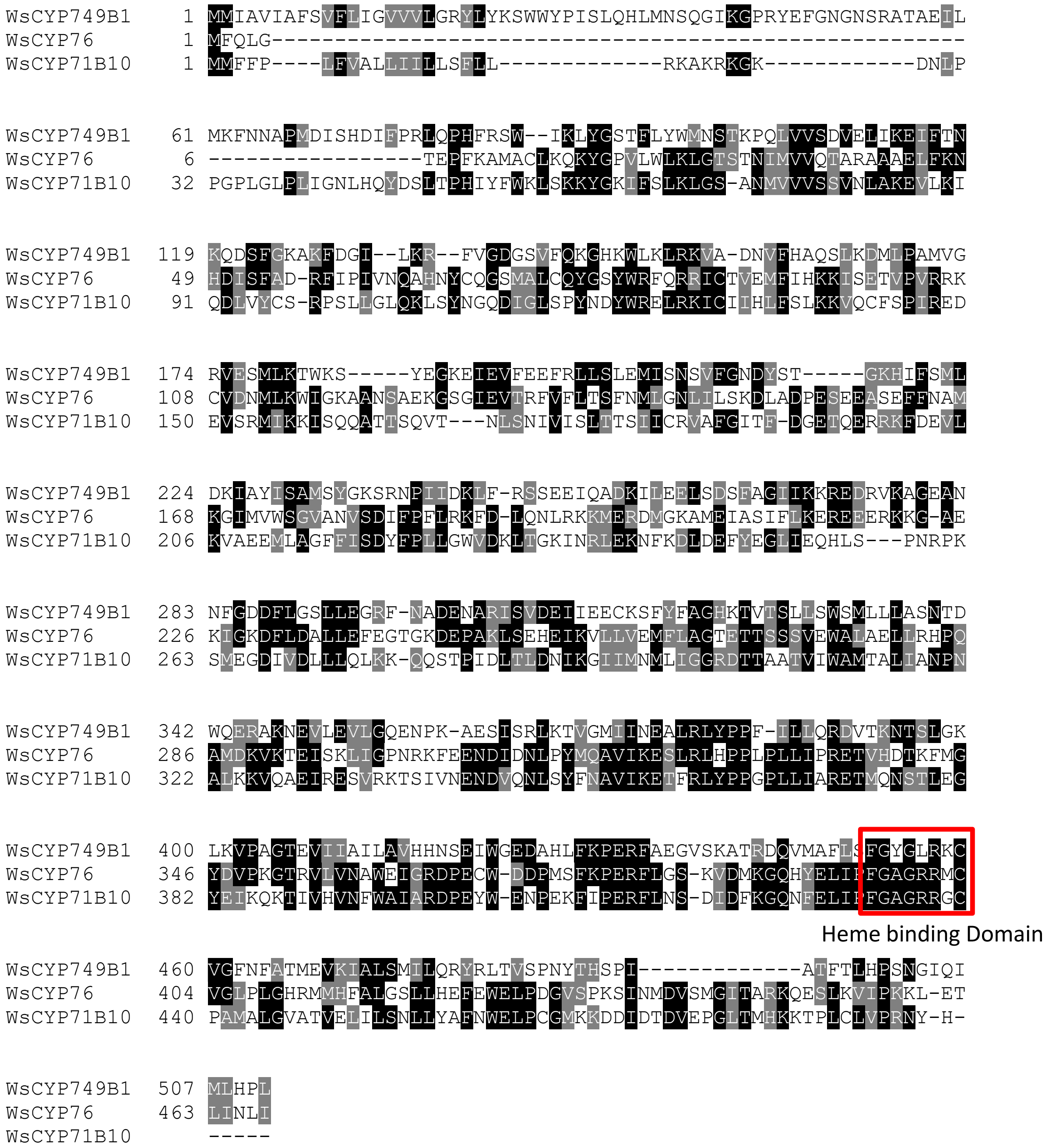

Figure S3: Multiple sequence alignment and box-shading of WsCYP450s

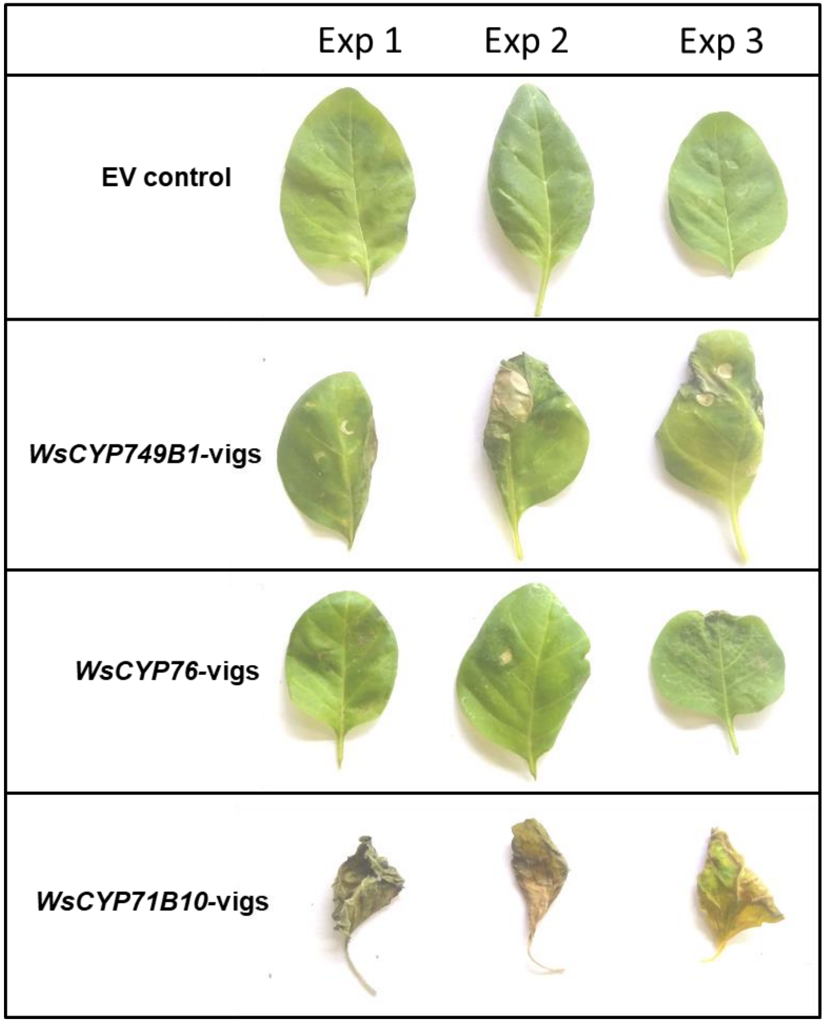

Figure S4: Phenotype of EV control and *WsCYP450*-vigs leaves of *W. somnifera* at 3 days post inoculation with *P. syringae* DC3000.

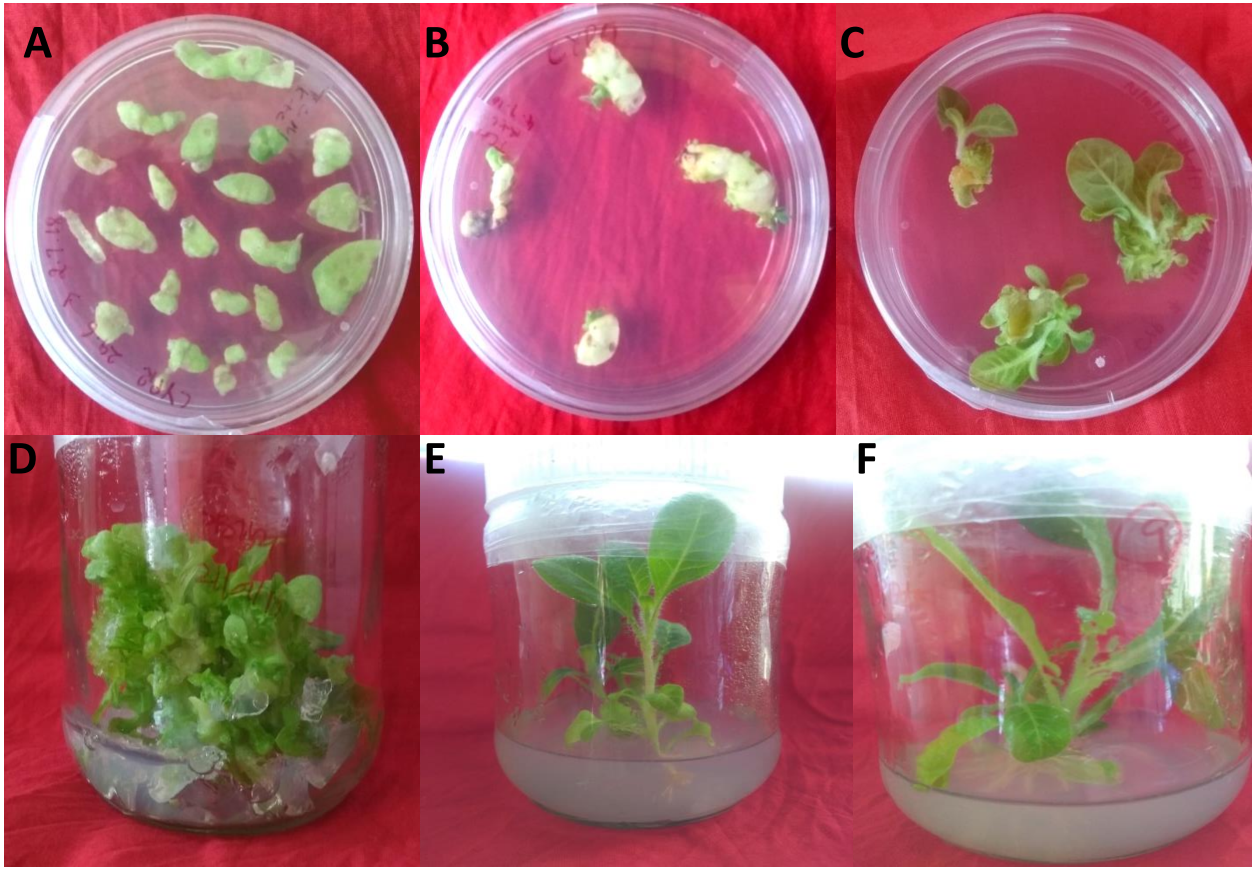

Figure S5: Representative figure showing generation of transgenic *N. tabacum* plants overexpressing *WsCYP450s*. A) Preculture of *N. tabacum* in tobacco callus initiation media (TCM) supplemented with 3% sucrose . B) Callus initiation with 50 mg/L kanamycin and 70 mg/L carbenicillin. C & D)Callus proliferation and shoot intitation with 50 mg/L kanamycin and 70 mg/L carbenicillin. E & F)Selection of nodal explants and multiple shoot induction. E & F) Root proliferation in half strength MS media.

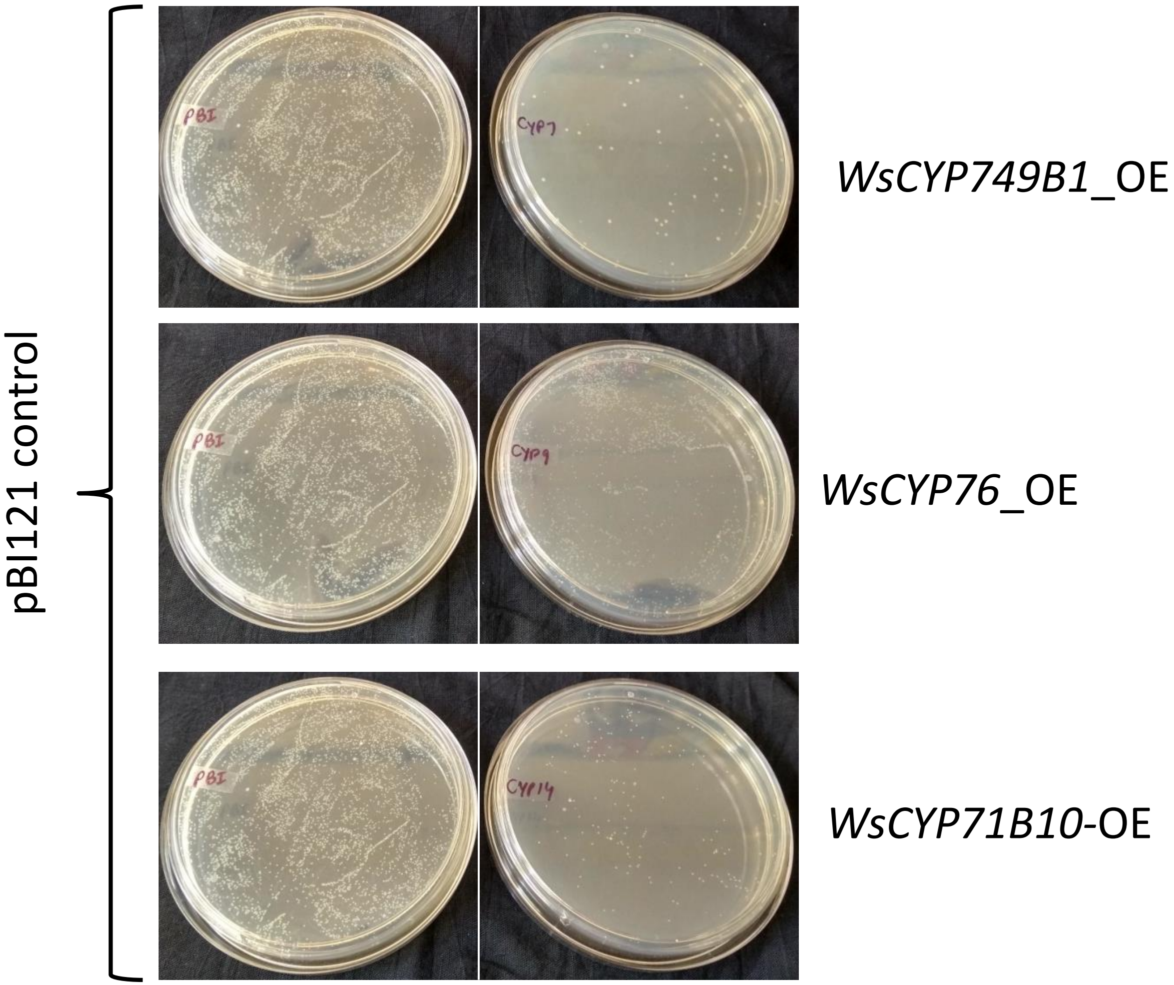

Figure S6: Representative plates from *P. syringae* DC3000 growth assay. Leaf discs from infected control and transgenic tobacco plants were collected from the zone of infiltration 3 days post inoculation. Leaf discs were homogenized using 10 mM $MgCl_2$ and the cfu/cm^2 was determined by plating serial dilutions of leaf extracts on *Pseudomonas*-specific agar plates. Plate on the left is of control and the same plate is used for comparison with all three transgenic lines.

Table S1: List of oligonucleotide primers used in the study ;Ws, *Withania somnifera*. RT_F and RT_R, forward and reverse primers used for RT-qPCR. pBI and pTRV2, primers used for pTRV2 and pBI121 cloning, respectively.

PRIMER	SEQUENCE	PURPOSE
WsCYP1-RT F	AAATGGGCTAAAGGCAGACTAACA	qRT- PCR
WsCYP1- RT R	CTTCCGTTTGGTGCAGGAA	qRT- PCR
WsCYP2- RT F	CTTTTCCCATTCGCTCGATTC	qRT- PCR
WsCYP2- RT R	CTGAAGGTACATGGCAAGGTTGA	qRT- PCR
WsCYP3- RT F	GGACAGAGTCAATGAGACGGTTAA	qRT- PCR
WsCYP3- RT R	GGAGCTGCTGAGTGCAAAGAA	qRT- PCR
WsCYP4- RT F	CCCTCAAAATCAAGCAACACATC	qRT- PCR
WsCYP4- RT R	CTCAAGGAACGCGAGGAAGA	qRT- PCR
WsCYP5- RT F	TCAGGGAAGGGAAAGCAAGA	qRT- PCR
WsCYP5- RT R	GCGGTTCCAACTTCGATCAA	qRT- PCR
WsCYP6- RT F	TGTTGCCTGGGCTTATTCG	qRT- PCR
WsCYP6- RT R	CAAAAGTGCCCTTAGTTTCTACAAGA	qRT- PCR
WsCYP749B1- RT F	TGCAAAATTGAAGCCAACACAT	qRT- PCR
WsCYP749B1- RT R	TCTAAAGCAACAAGGGACCAAGT	qRT- PCR
WsCYP8- RT F	CCTTCTATGTGTGGCCCATTTATC	qRT- PCR
WsCYP8- RT R	CTCATGCCAGCCCACTCTCT	qRT- PCR
WsCYP76- RT F	GGCTCCTATTGGCGGTTTC	qRT- PCR
WsCYP76- RT R	ACTGGCACCGTTTCACTGATC	qRT- PCR
WsCYP10- RT F	AGACAAGGAGGAGACATTTATAACAGAAG	qRT- PCR
WsCYP10- RT R	TGGTTTCATGAGCCGCAAA	qRT- PCR
WsCYP11- RT F	CAACTCCACTTTCCCCTTCATT	qRT- PCR
WsCYP11- RT R	TGCGTTGTGTTCTCTGACTAATAACA	qRT- PCR
WsCYP12- RT F	AAGCACTTGGGTTCTTGAGCAT	qRT- PCR
WsCYP12- RT R	CATGTTTGCAGCAGGAACAGA	qRT- PCR
WsCYP13-RT F	CATTTCTCATCGCTCTTCTCTTCTACT	qRT- PCR
WsCYP13- RT R	ACCTGGCGGGAAAACCTTT	qRT- PCR
WsCYP71B10- RT F	TTCCCCTCTTTGTAGCCCTTCT	qRT- PCR

PRIMER	SEQUENCE	PURPOSE
WsCYP71B10- RT R	GCAGATTGTCTTTCCCTTTCCTT	qRT- PCR
WsCYP15- RT F	CTTCAGGCTTTCGCAGGAGTT	qRT- PCR
WsCYP15- RT R	GGATTTATTCATTGCCGGAAGT	qRT- PCR
WsCYP16- RT F	TCCCAGACTCACTGCACAACTC	qRT- PCR
WsCYP16- RT R	GCACTCAACTTCGACCTGCAT	qRT- PCR
WsCYP17- RT F	GGATGGCTCCGATACAATGG	qRT- PCR
WsCYP17- RT R	CCTTTTTCATTACGTGTGGACTGT	qRT- PCR
WsCYP18- RT F	CAGCAAAATTGGCAAAAGAAGTG	qRT- PCR
WsCYP18- RT R	GACAATTTTTGCTGGCCAAGA	qRT- PCR
WsCYP19- RT F	TCTTTCCACTCCTTGTAGCCTTTC	qRT- PCR
WsCYP19- RT R	CAGATTGTTTTTGCCGCTCTT	qRT- PCR
WsCYP20- RT F	CCACTGTTCTCCCCTTCTACCAT	qRT- PCR
WsCYP20- RT R	ACGAGCTGTTGGTCCAAACC	qRT- PCR
WsCYP21- RT F	CACTCAGAAAGAAGCAGCAAACAT	qRT- PCR
WsCYP21- RT R	CTGTTGCTGAAAGTTGGCTTGT	qRT- PCR
WsCYP749B1 VIGS F	TCTAGATGCTGTTAAGAAGGCATGATG	pTRV2 cloning
WsCYP749B1VIGS R	CTCGAGGCATGGAAAACATTGTCAGCC	pTRV2 cloning
WsCYP76 VIGS F	TCTAGAGCTAGTAGAGAGATGGAATGG	pTRV2 cloning
WsCYP76 VIGS R	GGATCCGCTGCTTTCCCTATCCATTT	pTRV2 cloning
WsCYP71B10 VIGS F	TCTAGACATGATGTTCTTTCCCCTC	pTRV2 cloning
WsCYP71B10 VIGS R	CTCGAGGTAACTTGTGAAGTGGTGGC	pTRV2 cloning
WsCYP21 VIGS F	TCTAGAGCCTTGCCAAACAAACTTC	pTRV2 cloning
WsCYP749B1 PBI F	GGGATCCATGATGATAGCAGTAATAGCTTTTTCTG	pBI121 cloning
WsCYP749B1 PBI R	GGAGCTCTTACAGTGGATGAAGCATGATT	pBI121 cloning
WsCYP76 PBI F	GGGATCCATGTTTCAACTAGGAACCGAGC	pBI121 cloning
WsCYP76 PBI R	GGAGCTCCTAGATAAGATTGATGAGTGTCTC	pBI121 cloning
WsCYP749B1 PBI F	GCCCGGGATGATGTTCTTTCCCCTCT	pBI121 cloning
WsCYP749B1 PBI R	GGAGCTCCTAGTGATAATTTCTGGGAACAAGG	pBI121 cloning

Table S2: Names, accession numbers and function of plant enzymes belonging to CYP71, CYP76 and CYP749 used in phylogenetic tree.

CYP450	Plant (accession number)	Reported function
**PtCYP71B40**	*Populus trichocarpa* (AIU56748)	Converts aldoximes to nitriles which repel a generalist caterpillar
**AaCYP71AV1**	*Artemisia annua* (BAM68808)	Oxidation of amorpha-4,11-diene
**PaCYP71A1**	*Persea Americana* (P24465)	*trans*-cinnamic acid 4-hydrolase
**AtCYP71A13**	*Arabidopsis thaliana* (O49342)	Conversion of Indole-3-Acetaldoxime in Camalexin Synthesis
**AmCYP71AJ1**	*Ammi majus* (Q6QNI4)	Psoralen Synthase
**CrCYP71D12**	*Catharanthus roseus* (ACM92061)	Tabersonine 16-hydroxylase
**GmCYP71A10**	*Glycine max* (AAB94584)	Metabolism of phenylurea herbicides
**HmCYP71**	*Hyoscyamus muticus* (ABS00393)	Hydroxylation of sesquiterpenes
**SaCYP76F37**	*Santalum album* (AHB33941)	Santalene/bergamotene oxidase
**SaCYP76F42**	*Santalum album* (AHB33944)	Santalene/bergamotene oxidase
**CaCYP76B4,**	*Camptotheca acuminata* (AES93118)	Camptothecin biosynthesis
**CrCYP76B6**	*Catharanthus roseus* (Q8VWZ7)	Geraniol 10-hydroxylase
**SmCYP76B4**	*Swertia mussotii* (D1MI46)	Geraniol 10-hydroxylase
**OsCYP76M7,**	*Oryza sativa* (NP_001047185)	Unknown
**CaCYP749A22,**	*Capsicum annuum* (XP_016548653)	Unknown
**SiCYP749A22,**	*Sesamum indicum* (XP_011102246)	Unknown
**DzCYP749A22,**	*Durio zibethinus* (XP_022722687)	Unknown
**GhCYP749A22,**	*Gossypium hirsutum* (XP_016703986)	Unknown
**QsCYP749A22,**	*Quercus suber* (XP_023924616)	Unknown
**ItCYP749A22,**	*Ipomoea triloba* (XP_031101439)	Unknown
**HbCYP749A22,**	*Hevea brasiliensis* (XP_021646524)	Unknown
**CsCYP749A22,**	*Camellia sinensis* (XP_028104020)	Unknown

Table S3: Prediction of sub-cellular localization of WsCYP450s using different software tools

Prediction program	WsCYP749B1	WsCYP76	WsCYP71B10
iPSORT	having a signal peptide, not targetted to mitochondria or chloroplast	having a signal peptide, not targetted to mitochondria or chloroplast	having a signal peptide, not targetted to mitochondria or chloroplast
WoLF PSORT	Integral membrane protein, Endoplasmic reticulum	Integral membrane protein, Endoplasmic reticulum	Integral membrane protein, Golgi
Predotar	Endoplasmic reticulum	Endoplasmic reticulum	Endoplasmic reticulum
TargetP	Secretory pathway, i.e. the sequence contains SP, a signal peptide	Secretory pathway, i.e. the sequence contains SP, a signal peptide	Secretory pathway, i.e. the sequence contains SP, a signal peptide
ChloroP	No chloroplast transit peptide	No chloroplast transit peptide	No chloroplast transit peptide

Table S4. Possible involvement of WsCYP450s in withanolide biosynthetic steps based on observations made *in planta* silencing and overexpression studies.

Gene	Utilizes as it's content is increased in VIGS	Utilizes as it's content is decreased in OE	Forms as it's content is reduced in VIGS	Forms as it's content is increased in OE
WsCYP749B1	Withaferin A	12-deoxywithstromonolide	Withanolide A	Withanolide A
	-	-	-	Withanolide B
WsCYP76	-	12-deoxywithstromonolide	Withanolide A	-
WsCYP71B10	-	Withanolide A	Withanolide B	-
